# Assessment of Arf6 Deletion in PLB-985 Differentiated in Neutrophil-Like Cells and in Mouse Neutrophils: Impact on Adhesion and Migration

**DOI:** 10.1155/2020/2713074

**Published:** 2020-04-07

**Authors:** Jouda Gamara, Lynn Davis, Emmanuelle Rollet-Labelle, Tsunaki Hongu, Yuji Funakoshi, Yasunori Kanaho, Fawzi Aoudjit, Sylvain G. Bourgoin

**Affiliations:** ^1^Division of Infectious Disease and Immunology, CHU de Quebec Research Center, Quebec, QC, Canada G1V 4G2; ^2^Faculty of Medicine, Laval University, Quebec, QC, Canada G1V 0A6; ^3^Department of Physiological Chemistry, Faculty of Medicine and Graduate School of Comprehensive Human Sciences, University of Tsukuba, 1-1-1 Tennohdai, Tsukuba 305-8575, Japan

## Abstract

Chemoattractant sensing, adhesiveness, and migration are critical events underlying the recruitment of neutrophils (PMNs) to sites of inflammation or infection. Defects in leukocyte adhesion or migration result in immunodeficiency disorders characterized by recurrent infections. In this study, we evaluated the role of Arf6 on PMN adhesion *in vitro* and on migration to inflammatory sites using PMN-Arf6 conditional knockout (cKO) mice. In PMN-like PLB-985 silenced for Arf6 fMLP-mediated adhesion to the *β*2 integrin ligands, ICAM-1 and fibrinogen or the *β*1/*β*2 integrin ligand fibronectin was significantly reduced. Furthermore, overexpression of wild-type Arf6 promoted basal and fMLP-induced adhesion to immobilized integrin ligands, while overexpression of the dominant-negative Arf6 has the opposite effects. Using the *Elane-Cre* deleting mouse strains, we report that the level of Arf6 deletion in inflammatory PMNs isolated from the dorsal air pouches was stronger when compared to naïve cells isolated from the bone marrow. In PMN-Arf6 cKO mice, the recruitment of PMNs into the dorsal air pouch injected with LPS or the chemoattractant fMLP was significantly diminished. Impaired cell migration correlated with reduced cell surface expression of CD11a and CD11b in Arf6 cKO PMNs. Our results highlight that Arf6 regulates the activity and possibly the recycling of PMN integrins, and this compromises PMN migration to inflammatory sites.

## 1. Introduction

Polymorphonuclear neutrophils (PMNs) are generated from hematopoietic stem cells located in the bone marrow (BM) [1]. They are key cells in the innate immune system involved in the first line of defense against pathogens. Upon sensing danger signals and inflammatory mediators, PMNs start rolling along the blood vessel wall. Following firm arrest and transmigration through the vascular endothelium, PMNs migrate to the inflammatory sites. All of these processes involve molecular mechanisms like delivery of cell adhesion molecules and activation of PMN integrins [2, 3]. Integrins are expressed in all mammalian cells. Dynamic processes dependent on the functions of various Rab and Arf small GTPases regulate integrin cell surface expression through the endosomal recycling pathways and intracellular signalling-mediated ligand binding activity [4]. The *β*2 integrins LFA-1 and Mac-1 are the major integrins regulating leukocyte-endothelium interactions that are required for PMN extravasation and bacterial killing. Mutations in the common integrin *β*2 chain CD18 result in leukocyte adhesion deficiency type I, a rare disease characterized by leukocytosis and recurrent infections [5–8]. However, the PMNs express other integrins such as *α*4*β*1, *α*5*β*1, and *α*v*β*3 [9].

The correct timing of events leading to PMN activation is regulated through the integration of external signals picked up by transmembrane receptors that initiate numerous intracellular signalling pathways controlled by various G proteins including small GTPases [10]. Small GTPases switch into the active and inactive conformations when bound to guanosine triphosphate (GTP) and to guanosine diphosphate (GDP), respectively. GTPase-Activating Proteins (GAPs) and Guanine Nucleotide Exchange Factors (GEFs) specific for their small GTPases regulate this cycle [11]. These small GTPases, including those of the Arf family, such as Arf6, play roles in mediating integrin outside-in and inside-out signals and are critical components regulating PMN adhesion dynamics and migration [10, 12]. Arf6 differs from other Arfs in that it localizes specifically to the plasma membrane and endosomal compartments, which is thought to stem from the individual protein environment [13].

Arf6 has been implicated in different signalling pathways and cellular functions such as remodeling of the actin cytoskeleton and phagocytosis [14–16]. Overexpression of Arf6 or of its regulators in cancer cells suggests an important role in cell adhesion, migration, and invasive behaviour [17, 18]. The expression of Arf6 in PMNs and PMN-like cells (HL-60 or PLB-985 myeloid leukemia cells) has been previously reported [19, 20]. Arf6 plays roles in *N*-formyl-Methionyl-Leucyl-Phenylalanine (fMLP) receptor-mediated downstream signalling pathways leading to phospholipase D (PLD) activation and superoxide production in PMNs or differentiated PLB-985 cells [19, 20]. Several members of the cytohesin (CTH) family of Arf-GEFs are expressed by PMNs [10, 20–23]. Pharmacological inhibition of CTH-1 with SecinH3 or silencing of CTH-1 inhibited fMLP-mediated membrane translocation of Arf6 and Arf1 and prevented Arf6 activation in PMNs [20]. Recent studies suggest that CTH-1 differentially regulates activation of the leukocyte *β*2 integrins LFA-1 and Mac-1 [23, 24].

The impact of Arf6 knockdown on PMN adhesion and migration is yet to be investigated. The depletion of Arf6 in terminally differentiated human PMNs is not possible, and the knockout of the Arf6 gene in mice results in embryonic lethality [25]. To circumvent these issues, we silenced Arf6 in PMN-like PLB-985 cells to evaluate adhesion to immobilized integrin ligands *in vitro*, and we generated PMN-specific Arf6 conditional knockout (cKO) mice to assess PMN trafficking in *vivo*. The data suggest that Arf6 regulates PMN adhesion to the integrin ligands fibronectin, fibrinogen, and ICAM-1. We also analyzed the phenotypes of PMN-Arf6 cKO mice. Western blotting confirmed the depletion of Arf6 in mouse PMNs. Arf6 depletion reduced inflammatory stimulus-mediated PMN recruitment into the mouse air pouches and was associated with reduced cell surface expression of the *β*2 integrins LFA-1 and Mac-1.

## 2. Materials and Methods

### 2.1. Reagents

RPMI 1640, foetal bovine serum (FBS), Hank's balanced salt solution (HBSS), phosphate-buffered saline (PBS), and EDTA were purchased from Wisent (St-Bruno, QC, Canada). Calcein-AM was purchased from Calbiochem (San Diego, CA, USA). Dibutyryl cyclic AMP (dbcAMP), *N*-formyl-Methionyl-Leucyl-Phenylalanine (fMLP), dimethyl sulfoxide (DMSO), lipopolysaccharide (LPS) from *Escherichia coli* O26:B6, and fibrinogen were purchased from MilliporeSigma (Oakville, ON, Canada). ICAM-1 and fibronectin were purchased from R&D Systems Inc. (Minneapolis, MN, USA). Percoll was purchased from GE Healthcare (Baie-D'Urfé, QC, Canada). Recombinant murine Granulocyte-Macrophage Colony-Stimulating Factor (GM-CSF) and recombinant murine TNF-*α* were purchased from Bio Basic Inc. (Markham, ON, Canada) and PeproTech (Rocky Hill, NJ, USA), respectively. The synthetic *N*-myristoylated Arf6 (amino acids 2-13) and *N*-myristoylated scramble peptides were obtained from CanPeptide Inc. (Pointe-Claire, QC, Canada). Annexin V-FITC and 7-amino-actinomycin D (7AAD) were purchased from BD Biosciences (San Jose, CA, USA).

### 2.2. Antibodies

V450-conjugated rat anti-mouse CD11b (clone M1/70), PE-conjugated rat anti-mouse CD11a (clone 2D7), and APC-conjugated rat anti-mouse Ly6G (clone 1A8) mAb were purchased from BD Biosciences (Mississauga, ON, Canada). Polyclonal anti-PI3 kinase p85 was purchased from Upstate Biotechnology (Temecula, CA, USA). The anti-actin antibody was bought from MilliporeSigma (Markham, ON, Canada). Anti-Arf6 mouse monoclonal IgG2b (clone 3A-1) was obtained from Santa Cruz Biotechnology (Dallas, TX, USA).

### 2.3. Isolation of Human Neutrophil PMNs

Venous blood was collected from healthy adult volunteers in an isocitrate anticoagulant solution. PMNs were prepared as described previously [26]. Leukocytes were purified by sedimentation in 2% Dextran T500 to remove the erythrocytes before centrifugation on Ficoll-Paque cushions to eliminate the mononuclear cells. Contaminating erythrocytes were removed by a 20 s hypotonic lysis in water, and PMNs were resuspended in HBSS, pH 7.4, containing 1.6 mM Ca^2+^ but no Mg^2+^.

### 2.4. Apoptosis Measurements

Human PMNs were incubated with the indicated concentration of synthetic *N*-myristoylated peptides for 1 h at 37°C. Apoptosis was monitored by Annexin V-FITC staining (5 *μ*L) and flow cytometry analysis using a FACSCalibur cytometer (BD Biosciences, Mississauga, ON, Canada).

### 2.5. Cell Culture

PLB-985 cells were grown in RPMI 1640 supplemented with 10% heat-inactivated FBS and differentiated to PMN-like cells by the addition of 0.3 mM dbcAMP for 3 days as described previously [23, 24].

### 2.6. Cell Transfection with siRNA and Arf6 Vector Constructs

PLB-985 cell transfections were carried out on a Nucleofector II (Amaxa Biosystems, Cologne, Germany) after 24 h of differentiation with 0.3 mM dbcAMP with 3 *μ*g of Arf6 siRNA (Invitrogen, Carlsbad, CA, USA) or nonsilencing siRNA duplexes (Qiagen, Toronto, ON, Canada), using program U-002 and our in-house transfection buffer as described previously [26]. After nucleofection, cells were transferred rapidly to the differentiation medium (RPMI 1640 supplemented with 10% heat-inactivated FBS and 0.3 mM dbcAMP). Cell functions were monitored at 48 h postnucleofection.

The Arf6 coding sequence was subcloned in frame with EGFP into a pEGFP-N3 expression vector using the EcoRI/BglII restriction sites. The Arf6 (Q67L) mutant defective in GTP hydrolysis and the Arf6 (T27N) mutant defective in GTP binding were generated by PCR amplification, and nucleotide changes were verified by direct sequencing. Differentiated PLB-985 cells were transfected as described above using 20 *μ*g of plasmid DNA. The fluorescent GFP^+^ live cells were sorted at 48 h posttransfection using a BM FACSAria II cell sorter. GFP^+^ and 7AAD^−^ cells were detected with 525/25 nm band-pass and 670 long-pass filters, respectively, using the 488 nm laser. Cell responses were monitored in both GFP^−^/7AAD^−^- and GFP^+^/7AAD^−^-differentiated PLB-985 cells (Supplementary Fig. 1 and Supplementary Table 1).

### 2.7. Adhesion to Extracellular Matrix Proteins

Ninety-six-well plates were coated overnight at 4°C either with 1 *μ*g/mL ICAM-1, 150 *μ*g/mL fibrinogen, or 50 *μ*g/mL fibronectin in NaHCO_3_ 0.1 M, pH 9.6. PMNs or differentiated PLB-985 cells were loaded with calcein-AM and distributed in the wells (5 × 10^5^ cells) before stimulation with 10^−6^ M fMLP for 30 min at 37°C in the dark. The adhered cells were lysed in H_2_O and their number estimated using a standard curve of lysates from calcein-labeled cells as described previously [23, 24].

### 2.8. Generation of PMN-Specific Conditional Knockout (cKO) Mice

The Arf6-floxed mice have been described previously [27, 28]. C57Bl/6 mice were from The Jackson Laboratory (Bar Harbor, ME, USA). Dr. Paul Kubes (U. of Calgary, Canada) provided the transgenic *Elane-Cre* mice expressing the *Cre* recombinase under the control of the PMN elastase (*Elane*) promoter [29]. *Elane-Cre* mice were backcrossed with C57Bl/6 mice. Mating the Arf6-floxed mice with the *Elane-Cre* mice generated PMN-Arf6 cKO mice ([30–33] and Supplementary Fig. 2). The mouse genotypes used in the study include wild-type C57Bl/6 (*Cre^−/−^*), heterozygous (*Cre^+/-^*), and homozygous (*Cre^+/+^*) *Cre* recombinase-expressing mice, Arf6-floxed mice (*flox^+/+^Cre^−/−^*), and PMN-Arf6 cKO mice heterozygous (*flox^+/+^Cre^+/-^*) or homozygous (*flox^+/+^Cre^+/+^*) for the *Cre* transgene. Age- (12- to 18-week-old) and sex-matched mice were used for *in vivo* and *in vitro* experiments. All experimental procedures were performed according to the guidelines of the Canadian Council on Animal Care and were approved by the CHU de Québec Committee on Animal Use and Housing.

### 2.9. Blood Cell Counting

Mouse mandibular vein blood drops were collected in EDTA-coated microtubes, and 10 *μ*L of blood was used to generate the complete blood formula using the scil Vet abc Plus+ hematology analyzer (scil animal care company Inc., Barrie, ON, Canada).

### 2.10. Purification of Mouse PMNs from the BM

Mouse PMNs were isolated from the BM of sex- and age-matched mice (12-18 weeks old). After euthanizing the mice, the BM from 3 to 5 mice was flushed from the dissected femur and tibia using a 27-gauge needle and a 1 mL syringe filled with HBSS supplemented with 10% FBS and 1 mM EDTA. BM cells were filtered through a 40 *μ*m cell sieve into a 50 mL conical tube, centrifuged (10 min, 1 700 rpm), resuspended in a 45% Percoll solution, and then layered onto a 3-step discontinuous gradient with 3 mL layers each of 55%, 65%, and 81% isotonic Percoll solutions. After centrifugation (30 min, 1600 g), PMNs were collected at the interface of the 81% and 65% Percoll layers. Cells were washed with HBSS and resuspended in HBSS containing 0.1% BSA. Cells were further purified by negative selection using the EasySep™ Mouse Neutrophil Enrichment Kit according to the manufacturer's protocol (STEMCELL Technologies Inc., Vancouver, BC, Canada).

### 2.11. Air Pouch Experiments

Dorsal air pouches were raised by subcutaneous injection of sterile air on days 0 and 3 as previously described [34]. On day 7, LPS (500 ng), fMLP (10^−7^ M), or the same volume of diluent (1 mL PBS) was injected into the air pouches. The mice were killed by CO_2_ asphyxiation after 4 h, and air pouches were washed twice with 0.9 mL HBSS containing 10% FBS and 5 mM EDTA. Air pouch lavage fluids were centrifuged at 3500 rpm, and pellets were resuspended in 2 mL lavage solution for cell counting using the Moxi Z mini automated cell counter (ORFLO, Ketchum, ID, USA).

### 2.12. RT-PCR Analyses

PMNs from BM of 5 mice were purified, and total RNA was extracted (~10^7^ cells) using the RiboZol RNA Extraction Reagent (Amresco, Solon, OH, USA) according to the manufacturer's protocol. RNA (1 *μ*g) was reverse transcribed using the RNA to cDNA EcoDry Premix (Takara Clontech, Mountain View, CA, USA) and Taq DNA Polymerase (New England Biolabs, Whitby, ON, Canada). GAPDH was used as an internal control for PCR amplification. Amplification conditions were as follows: 30 cycles at 95°C (denaturation, 30 s), 56°C (annealing, 30 s), and 68°C (extension, 30 s). Primer sequences were as follows: *Arf6* (5′-ATCCTGTACAAGTTGAAGCTGGGC-3′ and 5′-CATCCCACACGTTGAACTTGACG-3′) and *GAPDH* (5′-AACTTTGGCATTGTAGAAGG-3′ and 5′-ACACATTGGGGTTAGGAACA-3′).

### 2.13. Western Blotting

Proteins were resolved on a 7.5-20% gradient SDS-PAGE and transferred to the Immobilon-PSQ PVDF Membrane (Millipore, Etobicoke, ON, Canada). Western blots were performed using Arf6 (1/200) and actin (1/1000) or PI3 kinase p85 (1/1000) antibodies as an internal control of protein loading. Proteins were visualized with HRP-conjugated anti-mouse secondary Ab (1/20000) and the Western Lightning Plus ECL detection system (PerkinElmer, Woodbridge, ON, Canada).

### 2.14. Analysis of *β*2 Integrin Cell Surface Expression

Where indicated, BM PMNs were primed with GM-CSF (100 ng/mL) and TNF (100 ng/mL) for 20 min at 37°C and/or stimulated with fMLP for 15 min at 37°C before analysis of *β*2 integrin cell surface expression. PMNs obtained from the BM and from LPS- or fMLP-injected air pouches were resuspended in 100 *μ*L cold PBS and incubated with 1 *μ*L of anti-CD11a, CD11b, and Ly6G Abs (or isotype control IgG) for 30 min at 4°C. PMNs were then washed twice and resuspended in 100 *μ*L cold PBS, and *β*2 integrin cell surface expression was monitored as described previously [23, 24].

### 2.15. Statistical Analysis

Data are mean ± SEM of at least three independent experiments. Statistical analysis was conducted using the unpaired Student's *t*-test and one-way ANOVA. A value of *p* < 0.05 was considered statistically significant.

## 3. Results

### 3.1. Arf6 Regulates Adhesion of PLB-985 to Ligands for the *β*2 Integrins LFA-1 and Mac-1

**Figure 1:**
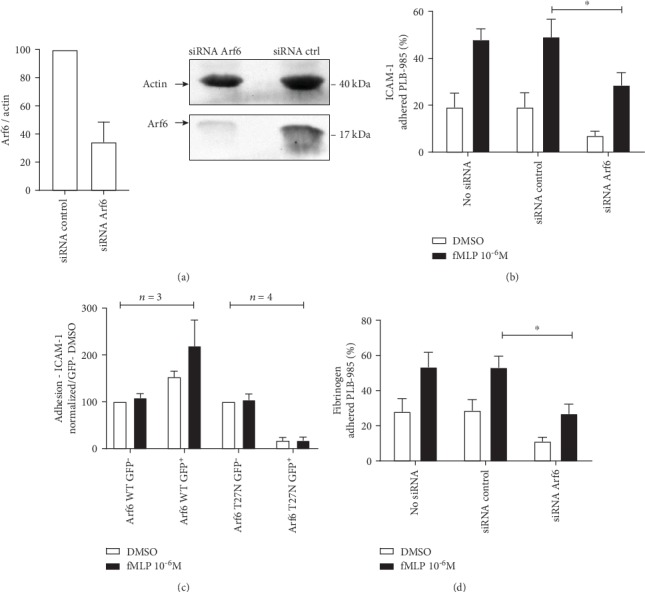
Arf6 regulates fMLP-induced PLB-985 adhesion to immobilized *β*2 integrin ligands. (a) Arf6 siRNA and negative control siRNA were transfected into PLB-985 cells as described in Materials and Methods. Arf6 expression was analyzed 48 h posttransfection by immunoblotting with anti-Arf6 and anti-actin (loading control) antibodies (*n* = 3 independent experiments). The left panel is from one representative experiment. (b) PMN-like PLB-985 silenced or not for Arf6 (48 h) were labeled with calcein-AM and stimulated with fMLP for 30 min to promote adhesion to immobilized ICAM-1 (*n* = 4 independent experiments performed in triplicate). (c) PMN-like PLB-985 were transfected with the various Arf6-GFP constructs for 48 h, GFP^+^ and GFP^−^ cells were sorted using GFP setting as previously described [23], and fMLP-mediated adhesion of calcein-labeled PMNs to ICAM-1 was evaluated (*n* = 3 or 4 independent experiments performed in triplicate). (d) PMN-like PLB-985 were transfected with Arf6-specific siRNA or siRNA control (48 h), were labeled with calcein-AM 30, and stimulated with fMLP for 30 min to promote adhesion to immobilized fibrinogen (*n* = 4 independent experiments performed in triplicate).

Pharmacological inhibition of Arf-GEF CTH-1 with SecinH3 in PMNs and silencing of CTH-1 in PMN-like cells were reported to reduce fMLP-induced responses, including activation and recruitment of Arf6 to the plasma membrane [20]. CTH-1 also coordinated activation of both *β*1 and *β*2 integrins [23, 24]. However, it is unclear whether dysregulation in integrin activation highlighted by inhibition of CTH-1 was due to impaired activation of Arf6 and mislocalization of Arf1 upon SecinH3 treatment and/or of another member of the cytohesin family, such as cytohesin-3 that is expressed in PMNs [20, 23]. Arf6 was silenced in PLB-985 cells as previously described [20]. As shown in Figure 1(a), siRNA-treated PLB-985 cells exhibited a ~65% decrease in the intracellular levels of Arf6 compared to cells transfected with a nonsilencing siRNA. We first investigated the impact of silencing Arf6 on cell adhesion to immobilized human ICAM-1, one of the adhesion molecules expressed on the surface of endothelial cells that promotes PMN adhesion to the vascular endothelium before extravasation and migration to sites of inflammation. Silencing of Arf6 in PLB-985 cells resulted in diminished basal adhesion and reduced fMLP-mediated adhesion to ICAM-1 by 42 ± 17% compared to cells transfected with the nonsilencing siRNA (Figure 1(b)).

As an alternative approach, we transiently overexpressed wild-type Arf6-GFP, dominant-positive Arf6-GFP (Q67L), and dominant-negative Arf6-GFP (T27N) in PLB-985 cells. Using this strategy, up to 50% of the cells were GFP positive (GFP^+^) (Supplementary Fig. 2). However, none of the constructs had any detectable effect on adhesion to ICAM-1 when mixed populations of GPP^+^ and GFP-negative (GFP^−^) PLB-985 cells were used (data not shown). To circumvent this problem, GPF^+^ cells were sorted by FACS. Compared to PLB-985 expressing wild-type Arf6-GFP, less viable cells (GFP^+^/7AAD^−^) expressing dominant-negative Arf6 were obtained after triaging (Supplementary Table 1). Furthermore, fMLP-induced adhesion to ICAM-1 was reduced compared to nonsorted PLB-985 cells (data not shown). However, basal and fMLP-mediated adhesion to ICAM-1 was enhanced in cells expressing wild-type Arf6-GFP (Figure 1(c)). In contrast, PLB-985 cells expressing dominant-negative Arf6 adhered poorly to immobilized ICAM-1 when compared to cells expressing wild-type Arf6-GFP, respectively (Figure 1(c)).

Whereas activation of *β*2 integrin LFA-1 required CTH-1 in PMNs, CTH-1 has been reported to restrain fMLP-mediated activation of *β*2 integrin Mac-1 in PMNs [23]. The molecular mechanisms by which CTH-1 differently regulates LFA-1 and Mac-1 integrins have not been investigated but may involve Arf6. To test this possibility, we evaluated the effect of silencing Arf6 on the adhesion of PLB-985 cells to fibrinogen, a ligand for Mac-1 integrin. Basal and fMLP-mediated adhesion to fibrinogen was decreased by ~50% in cells silenced for Arf6 when compared to PLB-985 cells treated with or without the silencer negative control siRNA (Figure 1(d)), thereby suggesting that CTH-1 does not restrain the activation of Mac-1 through Arf6 signalling.

As a third approach, we also attempted to inhibit Arf6 using the synthetic myristoylated Arf6 *N*-terminal peptide [35]. However, concentrations of Arf6 inhibitory peptide or control myristoylated scrambled peptide higher than 1 *μ*M were cytotoxic for PMNs (Supplementary Fig. 3). When used at 1 *μ*M, the Arf6 inhibitory peptide was without effect on fMLP-induced PMN adhesion to immobilized *β*2 integrin ligands (Supplementary Fig. 3).

### 3.2. Inhibition of Arf6 Signalling Reduces Cell Adhesion to Immobilized Fibronectin

**Figure 2:**
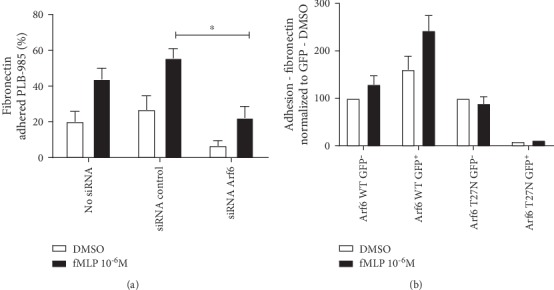
Arf6 regulates fMLP-induced PLB-985 adhesion to immobilized fibronectin. (a) PMN-like PLB-985 silenced or not for Arf6 (48 h) were labeled with calcein-AM and stimulated with fMLP for 30 min to promote adhesion to fibronectin (*n* = 4 independent experiments performed in triplicate). (b) PMN-like PLB-985 were transfected with the various Arf6-GFP constructs for 48 h. GFP^+^/7AAD^−^ and GFP^−^/7AAD^−^ cells were sorted using GFP setting as previously described [23], and fMLP-mediated adhesion of calcein-labeled PMNs to fibronectin was evaluated (Arf6 WT GFP^−^ and Arf6 WT GFP^+^*n* = 3; Arf6 T27N GFP^−^*n* = 2; Arf6 T27N GFP^−^*n* = 1 experiment performed in triplicate). Cell adhesion was evaluated, and data were normalized as described in Materials and Methods.

In the next series of experiments, we monitored the impact of silencing Arf6 on fMLP-mediated PLB-985 cell adhesion to the immobilized fibronectin, a *β*1/*β*2 integrin ligand. As shown in Figure 2(a), basal adhesion to fibronectin was reduced in Arf6-silenced PLB-985 cells. Arf6 knockdown also diminished the fMLP-mediated increase in cell adhesion by 62% when compared to cells transfected with control nonsilencing siRNA (Figure 2(a)). The impact of overexpressing Arf6-GFP (wild-type, dominant-positive, and dominant-negative) in PLB-985 cells on adhesion to fibronectin was also investigated. The transfection efficiency (40 to 50% GFP^+^ cells) was not sufficient to impact basal or fMLP-induced adhesion to fibronectin (data not shown and Supplemental Fig. 2). Therefore, PLB-985 cells overexpressing wild-type Arf6 and dominant-negative Arf6 were sorted by FACS. Overexpression of wild-type Arf6-GFP significantly increased basal and fMLP-mediated adhesion to fibronectin when compared to control GFP^−^ cells (Figure 2(b)). The adhesion of PLB-985 cells expressing dominant-negative Arf6 to fibronectin was drastically reduced when compared to cells expressing wild-type Arf6 (Figure 2(b)).

### 3.3. LFA-1 and Mac-1 Expression in BM-Extracted PMNs in the Arf6 cKO Mouse Model

**Figure 3:**
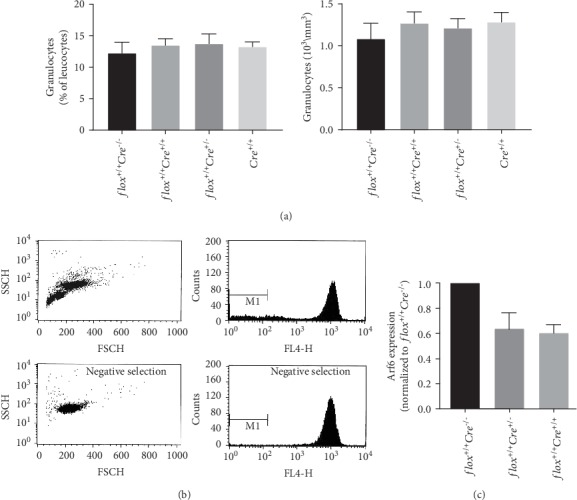
Characterization of PMN-Arf6 cKO mice and of BM-derived PMNs. (a) Peripheral blood granulocyte counts in PMN-Arf6 cKO mice bearing one (*flox^+/+^Cre^+/-^*) or two *Cre* alleles (*flox^+/+^Cre^+/+^*), in control Arf6-floxed (*flox^+/+^Cre^−/−^*) and control homozygous *Elane-Cre* (*flox*^−/−^*Cre^+/+^*) mice (*n* = 5 mice). Left (% of total leukocytes) and right (absolute number by mm^3^). (b) BM PMNs were purified from BMs using only Percoll gradient separation (upper panels) and negative enrichment (lower panels) as described in Materials and Methods. (c) Semiquantitative RT-PCR analysis of Arf6 mRNA levels in PMNs purified from BMs (*n* = 3-5 independent experiments).

PLB-985 cells are amenable to transfection and can be used as surrogate cells to study PMN functions. However, these cells do not possess secondary granules and express low levels of CD11b, and in contrast to PMNs, stimulation of dbcAMP-differentiated PLB-985 cells with fMLP does not increase cell surface expression of CD11b [36]. Since complete germline deletion of Arf6 is embryonic lethal [25], one possible avenue to study the function of Arf6 was to use specific mouse *Cre* strains to specifically knock out Arf6 in PMNs. To achieve these goals, Arf6-floxed mice were crossed with *Elane-Cre* mice that express *Cre* recombinase under the control of the granulocyte elastase promoter [29].  Figure 3(a) shows that peripheral blood granulocyte counts in PMN-Arf6 cKO mice bearing one (*flox^+/+^Cre*^+/-^) or two *Cre* recombinase transgenes (*flox^+/+^Cre^+/+^*) were identical to those of *Arf6*-floxed (*flox^+/+^*) and homozygote *Elane-Cre* (*Cre^+/+^*) mice.

**Figure 4:**
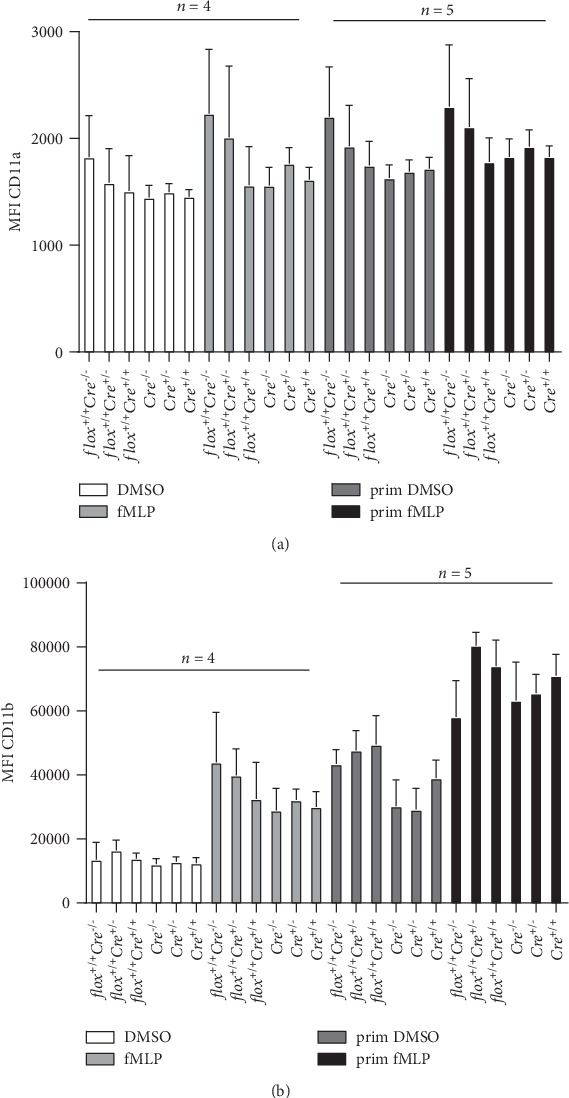
Cell surface exposure of CD11a and CD11b in control and stimulated BM PMNs. BM PMNs purified using a Percoll gradient and negative selection were treated without or with GM-CSF and TNF for 20 min before stimulation with fMLP for 15 min. Ly6G^+^ cells (PMNs) were gated to evaluate cell surface expression of CD11a (a) and CD11b (b) in all mouse strains (*n* = 4-5 independent experiments, 2 mice/genotype) (prim = GM-CSF and TNF priming).

PMNs from the BM were purified using a Percoll density gradient followed by immune-magnetic negative enrichment. Quantification of Ly6G^+^CD11b^+^ cells by FACS after the Percoll density gradient indicated that 84.4% of the cells were PMNs. PMN purity reached 99.9% after the negative enrichment step (Figure 3(b)). Monitoring Arf6 gene transcripts using qPCR indicated that Arf6 mRNA levels were decreased by ~36% and ~40%, respectively, in BM-derived PMNs from mice bearing one (*flox^+/+^Cre*^+/-^) or two (*flox^+/+^Cre*^+/+^) *Cre* recombinase alleles when compared to PMNs purified from control Arf6-floxed mice (Figure 3(c)). The data suggest that the complete deletion of Arf6 was not achieved in BM-derived PMNs of the *Elane-Cre* mouse strain. We next evaluated *β*2 integrin cell surface expression in BM-derived PMNs of various control and Arf6 cKO mice. Unprimed and GM-CSF-/TNF-primed PMNs were stimulated or not with fMLP before monitoring LFA-1 and Mac-1 by flow cytometry using CD11a and CD11b antibodies, respectively. Regardless of the mouse genotype, BM-derived PMNs expressed similar levels of LFA-1 (Figure 4(a)). Furthermore, stimulation with fMLP and priming with GM-CSF/TNF or their combination had no significant impact on the levels of LFA-1 cell surface expression (Figure 4(a)). In contrast, stimulation with fMLP or priming with GM-CSF/TNF enhanced surface expression of Mac-1. The combined effect of GM-CSF/TNF priming and fMLP stimulation on surface exposure of Mac-1 was additive. However, there was no difference in Mac-1 surface expression on BM PMNs of Arf6-cKO mice when compared to the *flox^+/+^* or the *Cre*^+/+^ strain (Figure 4(b)).

### 3.4. Recruitment of PMNs into the Air Pouch of Arf6 cKO Mice

**Figure 5:**
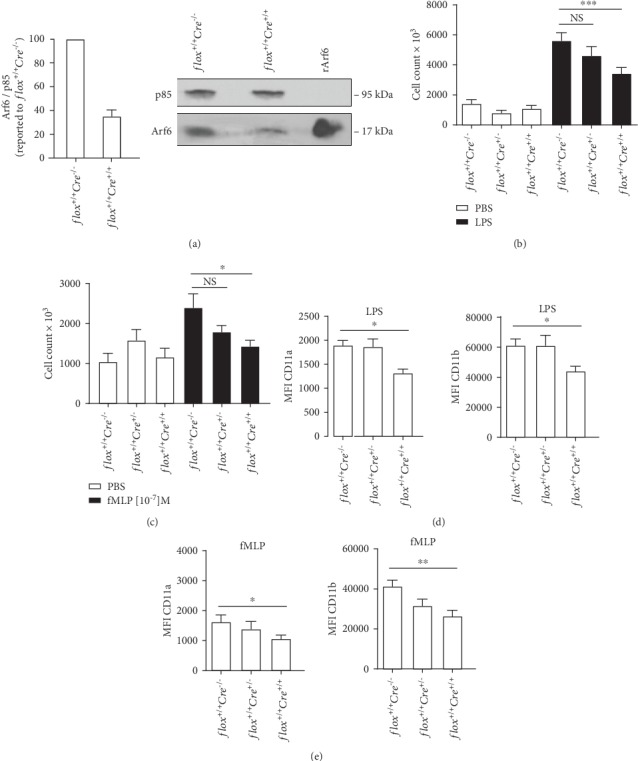
The deletion of Arf6 reduces PMN migration into the air pouches and cell surface expression of CD11a (LFA-1) and CD11b (Mac-1). (a) Arf6 expression by PMNs recruited into the mouse air pouches injected with LPS stimulation was analyzed by immunoblotting with Arf6 and PI3 kinase p85 alpha (p85, loading control) antibodies (left panel, *n* = 3). The right panel is a representative blot from 3 independent experiments with similar results. Recombinant Arf6 (rArf6) was used as a positive control. The amounts of PMNs recruited into the mouse air pouches injected with (b) LPS or (c) fMLP for 4 h were assessed (PBS: *n* = 6 mice; LPS: *n* = 30-46 mice; and fMLP *n* = 28-36 mice). (d) Ly6G^+^ cells were gated to evaluate cell surface expression of CD11a (left panel) and CD11b (right panel) in PMNs recruited into the mouse air pouches injected with LPS (*n* = 6 mice). (e) Cell surface expression of CD11a (left panel) and CD11b (right panel) in PMNs recruited into the mouse air pouches of injected with fMLP (*n* = 6-10 mice).

We also evaluated the levels of Arf6 in inflammatory PMNs that were recruited into the murine air pouch at 4 h postinjection of 500 ng LPS. Compared to control *flox^+/+^Cre^−/−^* mice, PMNs recruited into the air pouch of *flox^+/+^Cre^+/+^* mice showed a ~65% reduction in Arf6 protein expression (Figure 5(a)). The next experiments evaluated the impact of Arf6 deletion on the recruitment of PMNs initiated with the intrapouch injection of LPS or fMLP. Cells were collected at 4 h after injection of the inflammatory/chemotactic agents, at the time of the rapid rise and just before the peak of PMN recruitment in this mouse model [34, 37]. On the one hand, LPS-mediated recruitment of PMNs into the air pouch of *flox^+/+^Cre^+/+^* mice was significantly reduced by 39% and not affected in *flox^+/+^Cre*^*+/*-^ mice when compared to *flox^+/+^* controls (Figure 5(b)). Fewer PMNs were recruited in response to the intrapouch injection of fMLP. Nevertheless, fMLP-mediated PMN recruitment into the air pouch of *flox^+/+^Cre^+/+^* mice was reduced by 40% and there was no difference in PMN count between the *flox^+/+^Cre^+/-^* and *flox^+/+^* groups (Figure 5(c)). In contrast, PMN recruitment initiated by intrapouch injection of LPS or fMLP was not different between the wild-type and *Cre* transgenic groups (Supplementary Figs. 4A and 4B).

We next evaluated by flow cytometry the cell surface expression of LFA-1 and Mac-1 in air pouch PMNs. PMNs from wild-type, heterozygous, and homozygous *Cre* transgenic mice that migrated in response to intrapouch injection of LPS or fMLP showed similar levels of CD11a and CD11b expression (Supplementary Figs. 4C and 4D). Taken together, the data suggest that cell surface expression of *β*2 integrins is not altered in mice expressing the *Cre* transgene. However, PMNs recruited in response to injection of LPS or fMLP into the air pouch of *flox^+/+^Cre^+/+^* mice showed a lower level of cell surface CD11a when compared to the *flox^+/+^Cre^+/-^* and *flox^+/+^* animals (Figures 5(d) and 5(e), left panels). Cell surface expression of CD11b was also decreased in air pouch PMNs of *flox^+/+^Cre^+/+^* mice (Figures 5(d) and 5(e), right panels). Altogether, the data show that decreased PMN migration to sites of inflammation in Arf6 cKO mice bearing two *Cre* transgenes correlated with a stronger deletion of Arf6 and a decrease in cell surface expression of the *β*2 integrins.

## 4. Discussion

In cells, Arf6 localizes to the plasma membrane, the cytosol, and the endosomal compartments. Arf6 is known to regulate exocytosis, endocytosis, and recycling of various receptors through the endosomal recycling pathways and remodeling of the actin cytoskeleton [38]. These cellular processes impact cell movement, cell division, phagocytosis, and cholesterol homeostasis [39–42]. Cell migration is essential for the recruitment of leukocytes to sites of inflammation [5]. We previously reported that the Arf-GEF cytohesin-1 (CTH-1) controls Arf6 activation, phagocytosis, and *β*1- and *β*2-dependent adhesion of PMNs to endothelial cells or immobilized integrin ligands [23, 24]. However, our knowledge of the biological function of Arf6 in PMN integrin activation and recruitment to sites of inflammation remains sparse. Our results demonstrate that Arf6 regulates PMN adhesion to ICAM-1, fibrinogen, and fibronectin. Results obtained in PMN-specific Arf6 conditional knockout (PMN-Arf6 cKO) mice indicate that ablation Arf6 reduces chemotactic peptide- and LPS-mediated recruitment of PMNs into the air pouches. Reduced recruitment of PMNs into the air pouches correlated with reduced cell surface expression of the *β*2 integrins Mac-1 and LFA-1.

Arf6 regulates adhesion and phagocytosis by controlling cell surface expression of *β*1 integrins [43–45] and Fc*γ* receptors [46], respectively. By regulating the delivery of *β*1 integrin back to the plasma membrane via recycling endosomes, Arf6 coordinates cell adhesion, migration, and cancer cell invasion [17, 47–49]. Deletion of Arf6 in endothelial cells abolishes hepatocyte growth factor-stimulated *β*1 integrin recycling, and pharmacological inhibition of the Arf6-GEF suppresses tumour angiogenesis and growth [27]. In many cases, the inhibition of Arf6 activity or the silencing of Arf6 leads to intracellular retention and accumulation of *β*1 integrin and results in decreased cell adhesion and migration [27, 43, 50]. Although *α*4*β*1 and *α*5*β*1 integrins function as fibronectin receptors, it should be highlighted that fibronectin has also been reported to be a *β*2 integrin ligand [51–53]. Future studies should determine how Arf6 depletion affects the surface expression and recycling of *β*1 integrin in Arf6 cKO PMNs.

Several PMN functions are subject to regulation by Arf6 including degranulation [20], NADPH oxidase activity [19, 20], phagocytosis [23], and chemotaxis [23]. Knockdown of the Arf6 selective GEF CTH-1 had opposing effects on PMN integrin functions, inhibiting LFA-1-dependent adhesion and enhancing Mac-1-, *α*4*β*1-, and *α*5*β*1-dependent adhesion [23, 24]. However, the ability of CTH-1 to induce the activation of LFA-1 and to restrain integrin activities in PMNs cannot be attributed to Arf6 for two reasons. First, overexpression of Arf6 enhanced adhesion to ICAM-1, fibrinogen, and fibronectin. Second, dominant-negative Arf6 or silencing of Arf6 abrogated and diminished binding to the immobilized integrin ligands, respectively. Although inhibition of CTH-1 has no impact on the level of active Arf1 in response to fMLP, we previously showed that the recruitment of Arf1 to the membrane was significantly impaired [20]. It is not excluded that mislocalization of Arf1 could impact adhesion, cell polarization, and integrin clustering and activation during fMLP-induced PMN chemotaxis [44, 54–56].

Transgenic mice expressing a *Cre* recombinase under the control of tissue-specific promoters such as PMN gelatinase (*GE-Cre or Elane-Cre*), Lysozyme M (*LysM-Cre*), or MRP8 (*MRP8-Cre*) have been used to knock out a gene in PMNs [30]. *LysM-Cre* and *GE-Cre* generate conditional gene knockout in both PMNs and macrophages, whereas *MRP8-Cre* is more selective for PMNs. Furthermore, *Cre*-mediated deletion efficiency was partial and varied depending upon which tissue leukocytes were purified [30]. The deletion of gelatinase in PMNs impairs host immunity but has no impact on PMN recruitment to inflammatory sites [32]. In this study, *Elane-Cre* mice were used to induce Arf6 deficiency in PMNs. In Arf6-cKO mice, we observed that Arf6 mRNA levels in BM PMNs were decreased by about 40% and deletion efficiency was not different between mice having one or two *Cre* alleles. Furthermore, no significant reduction in the levels of endogenous Arf6 protein could be achieved in BM PMNs (data not shown). This most likely explains why fMLP-mediated cell surface expression of *β*2 integrins in controls and GM-CSF-/TNF-primed cells was not reduced in Arf6-cKO PMNs isolated from the BM. BM PMNs are naïve and in part immature cells. More efficient deletion of Arf6 in mature and noninflammatory PMNs cannot be excluded but is not easy to evaluate due to the low level of circulating PMNs in mice. Therefore, we evaluated the phenotype of PMNs recruited into the mouse air pouch following an injection of fMLP or LPS, two agonists which are known to activate receptors linked to Arf6 activation and signalling [19, 20, 35]. The level of endogenous Arf6 was depleted by about 65% in air pouch PMNs of Arf6 cKO mice expressing two *Cre* alleles. The deletion of Arf6 correlated with diminished recruitment of PMNs into the air pouches in response to fMLP or LPS and with reduced surface expression of LFA-1 and Mac-1 on inflammatory PMNs. Taken together, the data suggest that the deletion of Arf6 impacts the recruitment of PMNs to inflammatory sites through modulation of integrin cell surface expression and activity. Arf6 knockout is incomplete and possibly not uniformly depleted within PMNs. This could mean that the air pouch model of inflammation might indirectly favour the recruitment PMNs in which Arf6 is partially depleted. Furthermore, functional redundancy may exist among the Arf small GTPases [56].

The mechanisms by which Arf6 affects integrin activities remain to be investigated. Inhibition of Arf6 or silencing of Arf6 in PMNs reduces PLD activation and granule exocytosis [20]. Phosphoinositol-4-phosphate-5-kinase is also a downstream effector of Arf6 [57]. PLD and phosphoinositol-4-phosphate-5-kinase both contribute to the regulation of *β*1 and *β*2 integrin functions in various leukocytes [58–60]. In PMNs, intracellular LFA-1 resides in secondary granules and highly exocytosable gelatinase poor granules, whereas Mac-1 was identified in secretory vesicles, in gelatinase and secondary granules [61, 62]. Arf6 cKO PMNs represent an alternative model to understand how Arf6 regulates the dynamics of integrin recycling and their retention within the endosomes and granule/vesicle populations.

In conclusion, our data highlight that *Cre*-mediated deletion of Arf6 is more efficient in inflammatory PMNs compared to their naïve BM counterpart. The deletion of Arf6 reduces PMN adhesion to integrin ligands and migration to sites of inflammation. Arf6 cKO PMNs can be used to dissect how Arf6 impact the sensing of inflammatory mediators, cell polarity during migration, and other functional responses.

## Data Availability

The data used to support the findings of this study are included within the article and the supplementary information file. The data that are not shown in the article are available from the corresponding or the first author upon request.

## Supplementary Materials

Supplementary Materials Supplementary Table 1: the number of viable cells (7AAD^−^) GFP^+^ and GFP^−^ PLB-985 cells recovered following sorting after 48 h posttransfection. Supplementary Fig. 1: analyses of expression of the GFP constructs by FACS at 48 h posttransfection of PLB-985 cells. Supplementary Fig. 2: generation of PMN-Arf6 conditional KO mice and mouse genotypes used in this study. Supplementary Fig. 3: the *N*-myristoylated Arf6 inhibitory peptide induces apoptosis, and a noncytotoxic concentration of the inhibitory peptide does not inhibit fMLP-induced PMN adhesion. (A) Human PMNs were incubated with the *N*-myristoylated Arf6 inhibitory peptide and the scrambled peptide for 1 h at 37°C, and apoptosis was determined as described in Materials and Methods (*n* = 1, triplicate measurements). (B–D) Calcein-labeled PMNs were incubated with the inhibitory or the scrambled peptide (1 *μ*M) for 1 h before stimulation with fMLP (10^−6^ M) for 30 min. (B) Adhesion to immobilized ICAM-1 (*n* = 2 independent experiments performed in triplicate). (C) Adhesion to immobilized fibrinogen (*n* = 3 independent experiments performed in triplicate). (D) Adhesion to immobilized fibronectin (*n* = 2 independent experiments performed in triplicate). Supplementary Fig. 4: PMN migration into the air pouches and cell surface expression of the *β*2 integrins in air pouch PMNs of mice bearing one or two *Cre* transgenes. The amounts of PMNs recruited into the mouse air pouches injected with (A) LPS or (B) fMLP for 4 h were monitored (PBS *n* = 6 mice; LPS: *n* = 30-46 mice; and fMLP *n* = 28-36 mice). (C) Ly6G^+^ cells were gated to evaluate cell surface expression of CD11a (left panel) and CD11b (right panel) in PMNs recruited into the mouse air pouches injected with LPS (*n* = 6 mice). (D) Cell surface expression of CD11a (left panel) and CD11b (right panel) in PMNs recruited into the mouse air pouches injected with fMLP (*n* = 6-10 mice).
