# Supplementary material for: Assessment of Arf6 Deletion in PLB-985 Differentiated in Neutrophil-Like Cells and in Mouse Neutrophils: Impact on Adhesion and Migration
Source: Mediators Inflamm. 2020 Apr 7;2020:2713074. doi: 10.1155/2020/2713074 (PMC7166286; doi:10.1155/2020/2713074)
Supplement: Supplementary Materials — Supplementary Table 1: the number of viable cells (7AAD−) GFP+ and GFP− PLB-985 cells recovered following sorting after 48 h posttransfection. Supplementary Fig. 1: analyses of expression of the GFP constructs by FACS at 48 h posttransfection of PLB-985 cells. Supplementary Fig. 2: generation of PMN-Arf6 conditional KO mice and mouse genotypes used in this study. Supplementary Fig. 3: the N-myristoylated Arf6 inhibitory peptide induces apoptosis, and a noncytotoxic concentration of the inhibitory peptide does not inhibit fMLP-induced PMN adhesion. (A) Human PMNs were incubated with the N-myristoylated Arf6 inhibitory peptide and the scrambled peptide for 1 h at 37°C, and apoptosis was determined as described in Materials and Methods (n = 1, triplicate measurements). (B–D) Calcein-labeled PMNs were incubated with the inhibitory or the scrambled peptide (1 μM) for 1 h before stimulation with fMLP (10−6 M) for 30 min. (B) Adhesion to immobilized ICAM-1 (n = 2 independent experiments performed in triplicate). (C) Adhesion to immobilized fibrinogen (n = 3 independent experiments performed in triplicate). (D) Adhesion to immobilized fibronectin (n = 2 independent experiments performed in triplicate). Supplementary Fig. 4: PMN migration into the air pouches and cell surface expression of the β2 integrins in air pouch PMNs of mice bearing one or two Cre transgenes. The amounts of PMNs recruited into the mouse air pouches injected with (A) LPS or (B) fMLP for 4 h were monitored (PBS n = 6 mice; LPS: n = 30-46 mice; and fMLP n = 28-36 mice). (C) Ly6G+ cells were gated to evaluate cell surface expression of CD11a (left panel) and CD11b (right panel) in PMNs recruited into the mouse air pouches injected with LPS (n = 6 mice). (D) Cell surface expression of CD11a (left panel) and CD11b (right panel) in PMNs recruited into the mouse air pouches injected with fMLP (n = 6-10 mice). [file 2713074.f1.pdf]

Supplementary table

PLB-985 Arf6 WT GFP^{-} / 7AAD^{-} (10^{6} cells)	PLB-985 Arf6 WT GFP^{+}/ 7AAD^{-} (10^{6} cells)	PLB-985 Arf6 T27N GFP^{-} / 7AAD^{-} (10^{6} cells)	PLB-985 Arf6 T27N GFP^{+}/ 7AAD^{-} (10^{6} cells)
-	5.7	10.29	3.47
3.94	3.72	0.67	0.22
2.93	2.35	5.61	2.07
1.1	2.34	3.1	0.9

Supplementary table 1. The number of viable cells (7AAD^{-}) GFP^{+} and GFP^{-} PLB-985 cells recovered following sorting after 48h post-transfection.

Supplementary figures

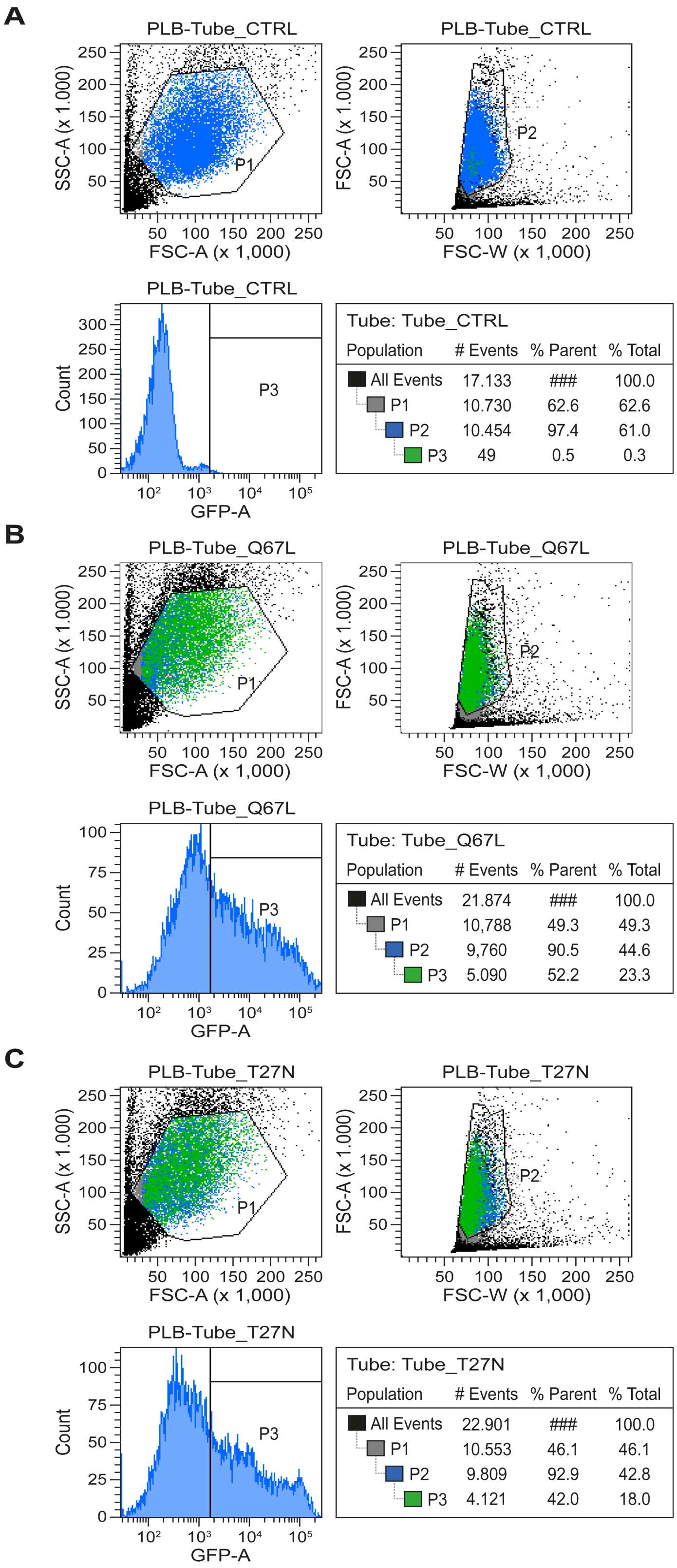

Supplementary Fig. 1. Analyses of expression of the GFP constructs by FACS at 48h post-transfection of PLB-985 cells.

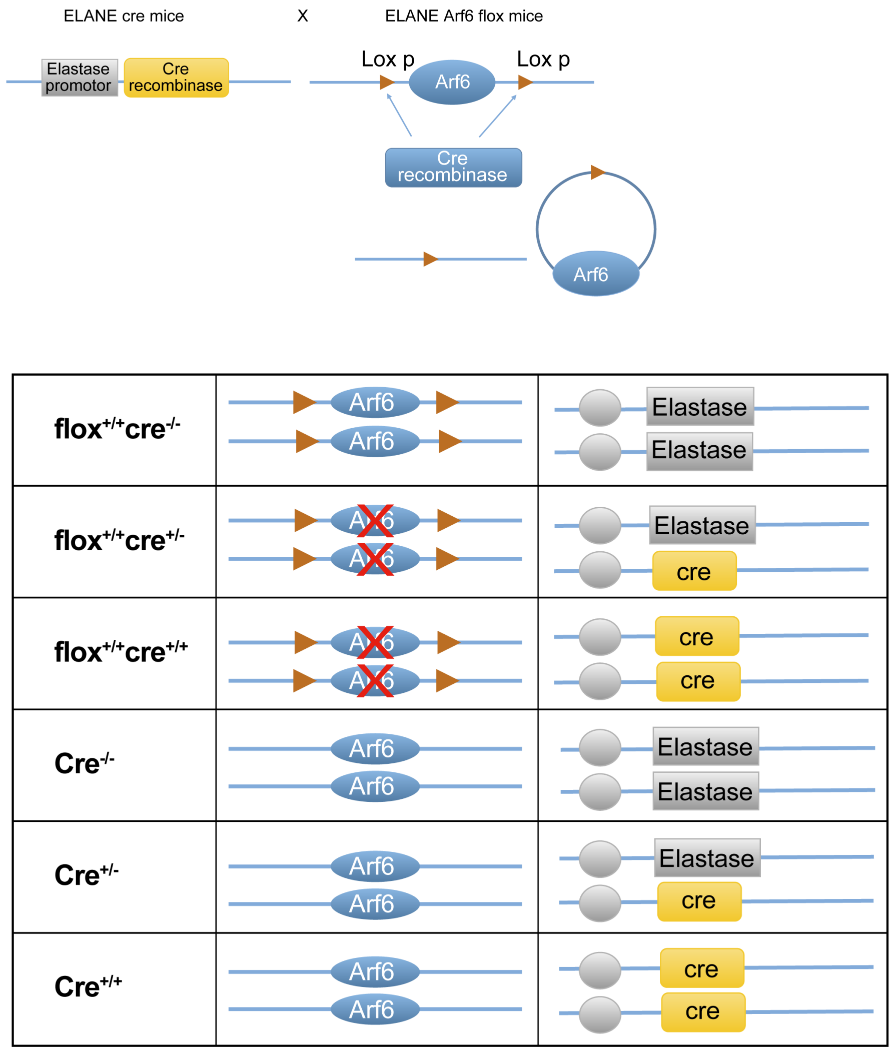

Supplementary Fig. 2. Generation of PMN-Arf6 conditional KO mice and mouse genotypes used in this study.

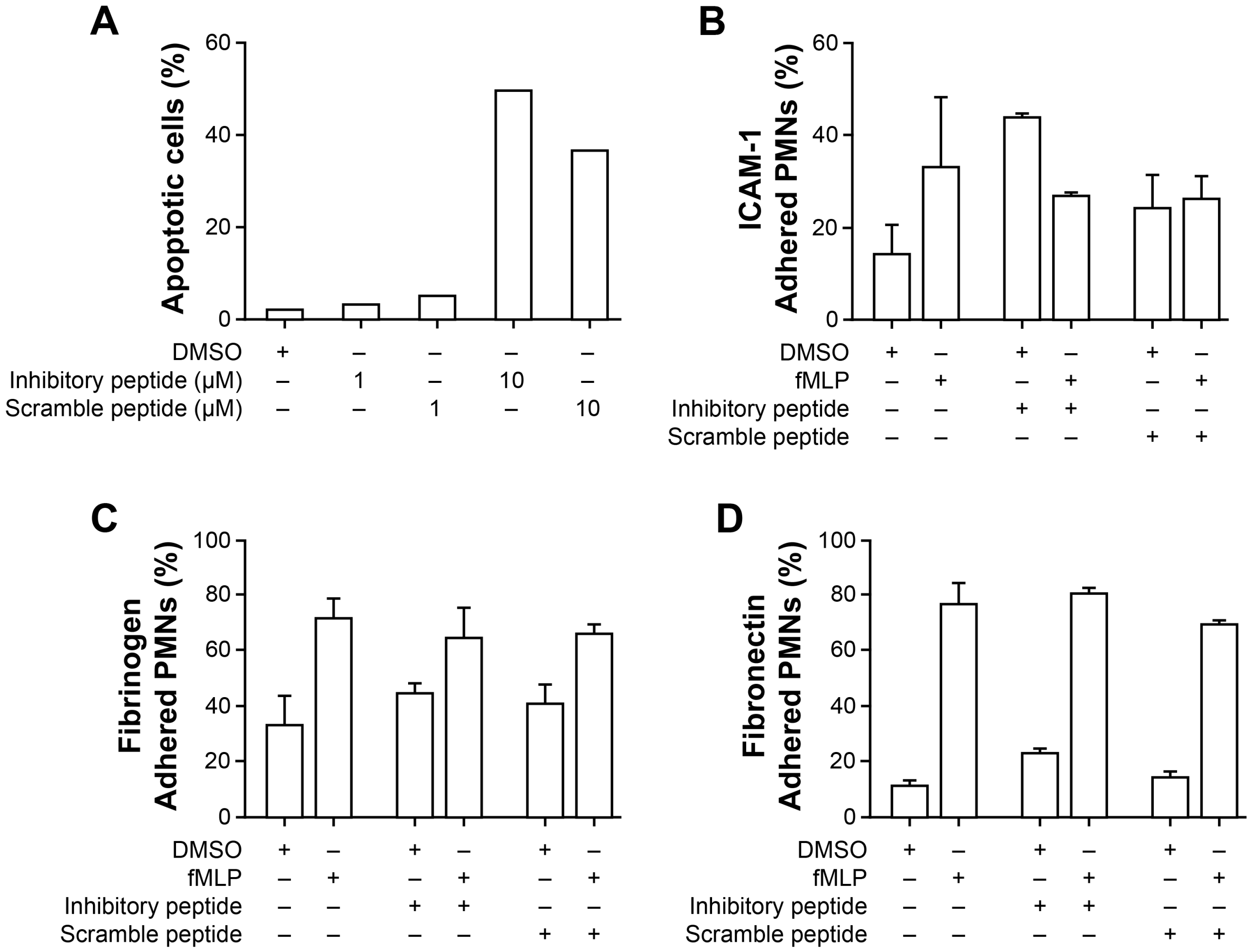

Supplementary Fig. 3. The N-myristoylated ARF6 inhibitory peptide induces apoptosis and a non-cytotoxic concentration of the inhibitory peptide does not inhibit fMLP-induced PMN adhesion. (**A**) Human PMNs were incubated with the N-myristoylated ARF6 inhibitory peptide and the scrambled peptide for 1h at 37°C and apoptosis was determined as described in Materials and Methods (n=1, triplicate measurements). (**B-D**) Calcein-labeled PMNs were incubated with the inhibitory or the scrambled peptide (1 μM) for 1h before stimulation with fMLP (10^{-6}M) for 30 min. (**B**) Adhesion to immobilized ICAM-1 (n=2 independent experiments performed in triplicate). (**C**) Adhesion to immobilized fibrinogen (n=3 independent experiments performed in triplicate). (**D**) Adhesion to immobilized fibronectin (n=2 independent experiments performed in triplicate).

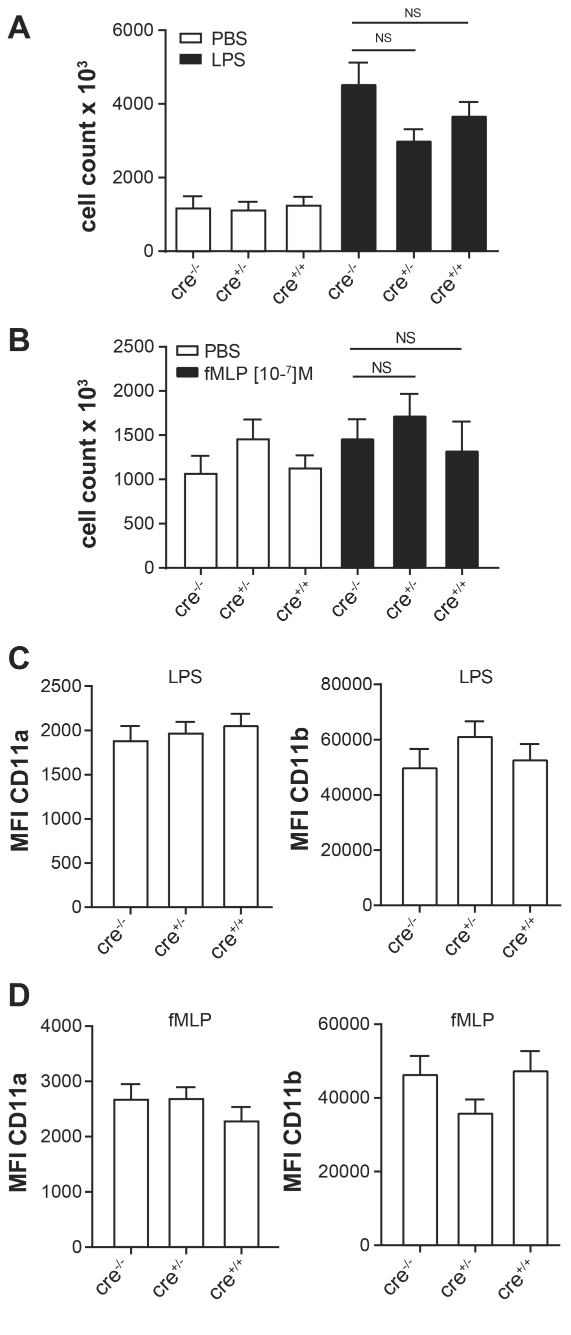

Supplementary Fig. 4. PMN migration into the air pouches and cell surface expression of the β2 integrins in air pouch PMNs of mice bearing one or two *Cre* transgenes. The amounts of PMNs recruited into the mouse air pouches injected with LPS (**A**) or fMLP (**B**) for 4h were monitored (PBS n=6 mice; LPS: n=30-46 mice; fMLP n=28-36 mice). (**C**) Ly6G$^+$ cells were gated to evaluate cell surface expression of CD11a (left panel) and CD11b (right panel) in PMNs recruited into the mouse air pouches injected with LPS (n=6 mice). (**D**) Cell surface expression of CD11a (left panel) and CD11b (right panel) in PMNs recruited into the mouse air pouches injected with fMLP (n=6-10 mice).
